# The types of endocrine cells in the pancreas of Sunda porcupine (*Hystrix javanica*)

**DOI:** 10.14202/vetworld.2016.563-567

**Published:** 2016-06-06

**Authors:** Teguh Budipitojo, Yuda Heru Fibrianto, Guntari Titik Mulyani

**Affiliations:** 1 Department of Anatomy, Veterinary Medicine Faculty, Gadjah Mada University, Yogyakarta, Indonesia; 2 Department of Physiology, Veterinary Medicine Faculty, Gadjah Mada University, Yogyakarta, Indonesia; 3 Department of Internal Medicine, Veterinary Medicine Faculty, Gadjah Mada University, Yogyakarta, Indonesia

**Keywords:** endocrine cell types, *Hystrix javanica*, immunohistochemistry, pancreas, Sunda porcupine

## Abstract

**Aim:**

To identify the types of endocrine cells in the pancreas of the Sunda porcupine (*Hystrix javanica*) and its immunolocalization.

**Materials and Methods:**

Five adult *H. javanica* were used without sexual distinction. The presences of endocrine cells (glucagon, insulin, somatostatin, and pancreatic polypeptide [PP]) in pancreatic tissues were detected using the avidin-biotin-peroxidase complex method.

**Results:**

The fusiform, round, and oval form endocrine cells were detected in the islets of Langerhans and exocrine parts. Most of the insulin cells were found in the central area, glucagon cells were identified in the central and peripheral areas, and somatostatin and PP cells were detected in the mantle area of the islets of Langerhans. Glucagon and somatostatin cells were also detected in smaller numbers of peripheral parts of the islet. In all of the islet parts, glucagon endocrine cells were most prevalent cell type and then, somatostatin, insulin, and PP. In the exocrine parts, PP, somatostatin, glucagon, and insulin endocrine cells were found in the inter-acinus part with moderate, moderate, a few and rare numbers, in that order. In the pancreatic duct, glucagon and somatostatin cells were found between epithelial cells in rare numbers.

**Conclusion:**

The pancreas of Sunda porcupine (*H. javanica*) contains four types of major pancreatic endocrine cells with approximately similar distribution patterns to the other rodents, except for abundant glucagon cells in the peripheral area of the islets of Langerhans.

## Introduction

The Sunda porcupine, *Hystrix javanica*, belonging to the genus Hystrix in the family Hystricidae is one of the Old World porcupines [1]. There are eight porcupine species of subgenera Acanthion, Hystric, and Thecurus within the genus Hystrix of porcupines. Sunda porcupine is one of the porcupine species belonging to the subgenus Acanthion, which endemic to Indonesia. This herbivore species is only found in Java, Bali, Sumbawa, Flores, Lombok, Madura, and Tanah Djampea island of Indonesia [2,3].

The pancreas is a composite gland consisting of exocrine and endocrine parts. The exocrine part consists of acini that secrete digestive enzymes into ducts. The endocrine part, the pancreatic islets (islets of Langerhans), consists of masses of endocrine cells embedded within the exocrine pancreas, where pancreatic hormones of insulin, glucagon, somatostatin, and pancreatic polypeptide (PP) are distributed into the whole body via blood vessels. The presence, distribution, and immunoreactivities of these regulatory hormones secreted by endocrine cells in the pancreas were well understood by histochemistry [4], immunofluorescence [5], and immunohistochemistry [6] methods. In other sides of those four pancreatic hormones, neurogenin 3 [7], galanin, and substance P [8] cells were also detected in the pancreas of vertebrates. The pancreas has been used as an important organ for endocrine research, especially in correlation with pancreatic disorders of diabetes [9]. At present, the distribution of gastrointestinal and pancreatic endocrine cells to be mentioned as a specific tool on a phylogenetic study since it has been known that the presences of immunoreactivity of the endocrine cells in the pancreas were diverse among species and feeding habits [10].

The recent studies on pancreas of porcupine have been conducted in crested porcupine (*Hystrix cristata*), which belongs to the subgenus Hystrix of the genus Hystrix [11,12]. The crested porcupine is extant in mainland Italy, Sicily, North Africa, and sub-Saharan Africa [13]. Unfortunately, the former researches on pancreas of *H. cristata* were limited on its basic morphology and localization of only 3 kind endocrine cells. There are no other data of pancreas on other porcupine species are available. In this research, the presences and semiquantitative distribution of all major endocrine cells types (insulin, glucagon, somatostatin, and PP) in the pancreas of Sunda porcupine, *H. javanica* which is endemic to Indonesia, were detected by employing the avidin-biotin-peroxidase complex (ABC) method.

## Materials and Methods

### Ethical approval

The present study was approved by the Ethics Committee for Preclinical Research of Gadjah Mada University, Yogyakarta, Indonesia (No.326/KEC-LPPT/IX/2015).

### Animals

Five pancreatic tissues of adult Sunda porcupines, *H. javanica*, about 67 cm in length, were purchased from an extreme food merchant in Tawangmangu, Central java, Indonesia, were used as samples. Pancreatic tissues of *H. javanica* were fixed for 24 h in Bouin’s solution, dehydrate in ethanol, cleared in xylene, and embedded in paraffin.

### Immunohistochemical staining

Pancreas sections were cut serially in 4-5 µm thicknesses and stained by hematoxylin and eosin for conventional histological evaluation. For immunohistochemical staining, tissue sections were deparaffinized in xylene and rehydrated in decreasing series of ethanol concentrations. After washing in phosphate-buffered saline, endogenous peroxidase activity was blocked by incubating the section in H_2_O_2_ in methanol for 15 min. Primary antibodies (Table-1) were applied overnight at 4° C. To prevent non-specific staining, sections were incubated with normal goat serum before incubation with the primary antibodies. The secondary antibody was applied for 45 min at room temperature. The immunoreactive site was visualized by the avidin-biotin-peroxidase complex method [14] and tris-HCL buffer containing 3,3 diaminobenzidine tetrahydrochloride. Section was counterstained with Mayer hematoxylin and examined with a conventional light microscope, and photomicrographs were taken with digital camera.

**Table-1:** Semiquantitative number of the endocrine cell types in the islets of Langerhans areas of the Sunda porcupine (*Hystrix javanica*).

Hormones	Area of the islets of Langerhans		

	Central	Mantle	Peripheral
Insulin	+++	++	-
Glucagon	+++	+	+++
Somatostatin	+	+++	+
Pancreatic polypeptide	±	±	++

-=Not detected, ±=Rare, +=A few, ++=Moderate, +++=Numerous

The specificity of the immunohistochemical staining was confirmed by the replacement of primary antibody with normal rabbit serum. The semiquantitative number of positive endocrine cell was graded subjectively into 4 classes as rare (±), few (+), moderate (++), and numerous (+++).

The presences of positif endocrine cell types were observed in 3 parts of the pancreas, (1) the islets of Langerhans, (2) the exocrine acini, and (3) the pancreatic duct. In addition, the presence and semiquantitatif number of endocrine cells types in the islets of Langerhans were distinguished into three locations: Central, mantle, and peripheral areas. The presence and semiquantitative number of endocrine cells types in the pancreatic duct were distinguished into two locations: inter-epithelial and sub-epithelial cells.

## Results

The research clarified the presence of four types pancreatic endocrine cells including insulin, glucagon, somatostatin, and PP in the islets of Langerhans, exocrine acini and pancreatic duct of *H. javanica*. Four types endocrine cells were detected in various pancreatic parts and areas with different semiquantitatif numbers as shown in Tables-1 and 2. The form of endocrine cells, which were detected in the pancreatic tissues of *H. javanica*, was fusiform, round, and oval.

**Table-2:** Semiquantitative number of the endocrine cells in the pancreatic duct and exocrine part of the Sunda porcupine (*Hystrix javanica*).

Hormones	Area of pancreatic ducts		Exocrine part

	Inter-epithelial cells	Sub-epithelial cells	
Insulin	-	-	++
Glucagon	±	-	±
Somatostatin	-	±	+
Pancreatic polypeptide	-	-	++

**Figure-1:**
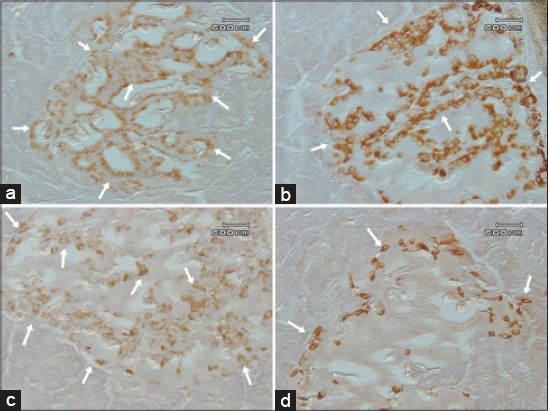
The insulin, glucagon, somatostatin and PP cells in the pancreatic islets of the Sunda porcupine. Insulin cells were demonstrated in the central and mantle regions (a), glucagon cells were mainly detected in the central and peripheral regions (b), somatostatin cells were dispersed throughout the whole central, mantle and peripheral regions (c), and PP cells were demonstrated in the central, mantle and peripheral regions (d) of pancreatic islets as indicated by arrows.

**Figure-2:**
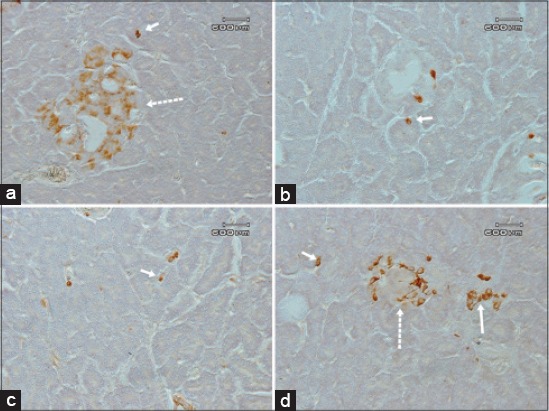
The insulin, glucagon, somatostatin and PP cells in the pancreatic acini of the Sunda porcupine. In the exocrine regions, insulin (a), glucagon (b), somatostatin (c) cell were detected between acinar cells, individually, while cluster of pancreatic polypeptide cells were detected between acinar cells (d) as indicated by solid arrows. Insulin (a) and pancreatic polypeptide cells were also detected in the small islets of Langerhans as indicated by discontinuous arrows.

-=Not detected, ±=Rare, +=A few, ++=Moderate,

The islets of Langerhans of *H. javanica* contain fusiform and round form insulin cells which located in the central and mantle areas with a numerous and moderate numbers, respectively, but not in the peripheral area (Figure-1a). The exocrine parts of *H. javanica* contain insulin cell types as an individual oval form cell which located between acinar cells with moderate number (Figure-2a). Insulin cell type was not found in the pancreatic duct.

**Figure-3:**
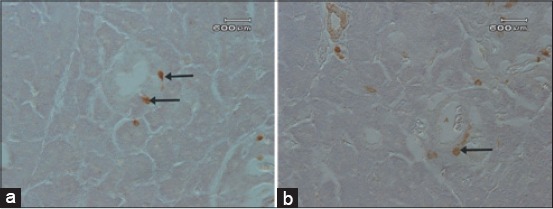
The glucagon and somatostatin cells in the pancreatic ducts of the Sunda porcupine. In the pancreatic duct, fusiform shaped glucagon cells were detected in the inter-epithelial cells of epithelial duct (a) and round shaped somatostatin cells were detected in the subepithelial cells of epithelial duct (b) as indicated by arrows.

Pancreatic islets of Langerhans consist of fusiform, oval, and round form of glucagon cell types, which were located in the central and peripheral areas with numerous numbers, but some of these cell types also can be identified in the mantle area in a few numbers (Figure-1b). Glucagon cell type was also detected in the exocrine parts, individually, and located between acinar cells with rare numbers (Figure-2b). The form of glucagon cell type is fusiform and round. The fusiform form of glucagon cell was also found in the inter-epithelial cells of the pancreatic duct in exocrine parts, with rare number (Figure-3a).

Somatostatin cells type was found in all areas of the islets of Langerhans of *H. javanica* with numerous numbers in mantle area and a few numbers in central and peripheral areas (Figure-1c). In these islets of Langerhans areas, the form of the somatostatin cells type was fusiform, oval, and round. The round and oval form of somatostatin cell type, individually, was found with a few number in the inter-acinar cells of exocrine acini (Figure-2c). The round form of the somatostatin cells type was also found in exocrine parts of sub-epithelial cells of the duct with a few numbers (Figure-3b).

The fusiform, oval, and round form of PP cells were mainly found in the peripheral area with a few numbers, and in the mantle and central areas with rare number of the islets of Langerhans (Figure-1d). A cluster of fusiform and round form of PP cells type were also found in inter-acinar cells of exocrine parts with moderate number (Figure-2d).

## Discussion

The presence of four main types of endocrine cells in the pancreas of the most exotic Indonesian endemic porcupine, the Sunda porcupine (*H. javanica*), has clarified base on the results of this research. They are insulin, glucagon, somatostatin, and PP of the endocrine cells types. The semiquantitatif number of endocrine cell types was grouped according to the pancreatic parts (endocrine islets, exocrine acini, and pancreatic duct parts) and the area of pancreatic islets (central, mantle, and peripheral areas). Specific spreading of endocrine cell types in the pancreatic tissues was also emphasized with references to other already known species.

In the pancreas of the Sunda porcupine, *H. javanica*, all of the four major endocrine cells types were mainly found in the endocrine parts but also detected in the exocrine parts. All these endocrine cells appeared mainly in fusiform and round form. Insulin is localized in the β-cells of the islets of Langerhans [15]. In the mammals, the distribution and the frequency of the endocrine cells in the pancreas of some rodents have been reported in the hamster [16], rat [17], mouse [4], gerbil [18], voles [19], and *H. cristata* [12]. Taken together, the previous reports established that insulin cells are commonly located in the central and mantle area of rodent pancreatic islets and that the other cells, glucagon, somatostatin, and PP cells, surrounded them. In agreement with previous reports on *H. cristata*, the present study of *H. javanica* found the insulin immunoreactive cells in the central and mantle areas with numerous and moderate number, respectively.

Glucagon is detected in the α-cells of the islets of the pancreas [20]. In *H. cristata*, glucagon cells were mainly found in mantle area of pancreatic islets, but some of these cells were also detected in the central area [12]. The distribution of glucagon cells in *H. cristata* is similar to that of other rodent pancreatic islets [4,16-19]. In contrast to the previous findings, the present study showed that in the islets of Langerhans of *H. javanica*, glucagon cells types were demonstrated mainly in the central and peripheral areas with numerous numbers, and only a few numbers of these cells were situated in the mantle area.

The types of endocrine cells in the islet of Langerhans that secrete glucagon, insulin, and somatostatin (A, B, and D cells, respectively) could exactly be distinguished from each other using immunohistochemical techniques [21]. Previous studies showed that in rodent pancreas, somatostatin cells were detected mainly in the most outer area [4,16-19] of islets of Langerhans. In the pancreas of a crested porcupine, *H. cristata*, the distribution of somatostatin differs from distributional patterns previously reported in other rodent species, which was mainly detected in mantle region [12]. The present study of Sunda porcupine agrees with the results on crested porcupine.

PP cell is the other type of endocrine cell that can be found in the pancreas. It has been known that the location differentiation of PP cells in the pancreas can easily be recognized among species, although the cells, if they occur, were always located in the peripheral area of the islets of Langerhans. PP cells were demonstrated in the peripheral area of the pancreatic islets of the hamster [16], rat [17], mouse [4], gerbil [18], and voles [19]. However, there are no available data on the distribution of PP cells in the pancreatic tissues of porcupines. The results of this research show in the first time that, in agreement with those of other rodent species, PP cells were detected in the islets of Langerhans of *H. javanica*, mainly in the peripheral area of the islets.

In the exocrine part of *H. javanica* pancreas, insulin, glucagon, and somatostatin cells were found individually, whereas PP cells were detected in a form of cluster cells located between acini. Moreover, glucagon and somatostatin cells were detected in the inter-epithelial cells and sub-epithelial cells of pancreatic ducts, respectively. Unfortunately, the report of Timurkan *et al*. [12] on pancreatic endocrine cell of the *H. cristata* did not mention about this features. The present result, however, the first evidence on the individual form of insulin, glucagon and somatostatin cells, and cluster cells feature of PP cells in the exocrine parts of porcupines species.

## Conclusion

The present research clarified the presence of four major endocrine cells, namely, insulin, glucagon, somatostatin, and PP cells in the islets of Langerhans and inter-acinar cells of the exocrine part. In pancreatic duct of *H. javanica*, glucagon cells were found in inter-epithelial cells, and somatostatin cells were detected in sub-epithelial cells. The endocrine parts of *H. javanica* pancreas contain of those four types endocrine cells with variation on the form, location, and number of the cells depend on the area of the islets of Langerhans but in common is approximately similar to that of other rodents. The research elucidated the exception on the specific location of glucagon cells in the peripheral area in islets of Langerhans of *H. javanica* which differ from others mammals.

## Authors’ Contributions

TB prepared proposal and managed the research implementation. TB, YHF and GTM carried out the sample collections and laboratory works for histology and immunohistochemistry. TB critically analyzed the histological and immunohistochemical data and prepared the manuscript. All authors read and approved the final manuscript.
